# Evaluating the Clinical and Physiological Effects of Long Term Ultraviolet B Radiation on Guinea Pigs (*Cavia porcellus*)

**DOI:** 10.1371/journal.pone.0114413

**Published:** 2014-12-17

**Authors:** Megan K. Watson, Adam W. Stern, Amber L. Labelle, Stephen Joslyn, Timothy M. Fan, Katie Leister, Micah Kohles, Kemba Marshall, Mark A. Mitchell

**Affiliations:** 1 Department of Veterinary Clinical Medicine, University of Illinois, Urbana, Illinois, United States of America; 2 Department of Pathobiology, University of Illinois, Urbana, Illinois, United States of America; 3 School of Veterinary Medicine and Biomedical Sciences, University of Nebraska-Lincoln, Lincoln, Nebraska, United States of America; Oxbow Animal Health, Murdock, Nebraska, United States of America; 4 PetSmart, Phoenix, Arizona, United States of America; Federal University of Rio de Janeiro, Brazil

## Abstract

Vitamin D is an important hormone in vertebrates. Most animals acquire this hormone through their diet, secondary to exposure to ultraviolet B (UVB) radiation, or a combination thereof. The objectives for this research were to evaluate the clinical and physiologic effects of artificial UVB light supplementation on guinea pigs (*Cavia porcellus*) and to evaluate the long-term safety of artificial UVB light supplementation over the course of six months. Twelve juvenile acromelanic Hartley guinea pigs were randomly assigned to one of two treatment groups: Group A was exposed to 12 hours of artificial UVB radiation daily and Group B received only ambient fluorescent light for 12 hours daily. Animals in both groups were offered the same diet and housed under the same conditions. Blood samples were collected every three weeks to measure blood chemistry values, parathyroid hormone, ionized calcium, and serum 25-hydroxyvitamin D_3_ (25-OHD_3_) levels. Serial ophthalmologic examinations, computed tomography scans, and dual energy x-ray absorptiometry scans were performed during the course of the study. At the end of the study the animals were euthanized and necropsied. Mean ± SD serum 25-OHD_3_ concentrations differed significantly in the guinea pigs (p<0.0001) between the UVB supplementation group (101.49±21.81 nmol/L) and the control group (36.33±24.42 nmol/L). An increased corneal thickness in both eyes was also found in the UVB supplementation compared to the control group (right eye [OD]: p<0.0001; left eye [OS]: p<0.0001). There were no apparent negative clinical or pathologic side effects noted between the groups. This study found that exposing guinea pigs to UVB radiation long term significantly increased their circulating serum 25-OHD_3_ levels, and that this increase was sustainable over time. Providing guinea pigs exposure to UVB may be an important husbandry consideration that is not currently recommended.

## Introduction

Guinea pigs (*Cavia porcellus*) are members of the order Rodentia and family Caviidae. They were native to the Andes and other mountainous regions of South America, where they were predominately used for food in countries such as Peru, Colombia, Venezuela, and Brazil [1]. At this time they are considered extinct in the wild [2]. In the United States, guinea pigs are popular pets. In a survey performed by the AVMA in 2006, there were over 1.3 million guinea pigs being kept as pets in the United States [3]. Guinea pigs are docile, responsive animals with a lively personality, and therefore make appealing pets for most households. Guinea pigs have also been used in research for over 400 years [4].

Current husbandry recommendations for guinea pigs are made with the goal of improving their health and longevity. However, at this time there are no specific lighting recommendations for guinea pigs other than providing a photoperiod of 12 hours to mimic the natural diurnal cycle [5]. This is interesting to note as these diurnal animals have evolved in a high altitude environment and would be expected to have increased exposure to ultraviolet B (UVB) radiation compared to animals at lower altitudes. Vertebrates can utilize UVB exposure as a method of generating endogenous vitamin D, an essential hormone. Whether exposure to UVB radiation is important for these animals relative to this function is not known.

Vitamin D is a circulating hormone that is important to homeostasis and normal physiology, including bone development, growth, neuromuscular function, reproduction, cardiovascular health, and immune function [6], [7]. Vitamin D deficiencies in humans and other vertebrates have been shown to cause rickets, osteomalacia, and reproductive failure [6], [7]. Not only do deficiencies directly lead to abnormal calcium metabolism, it is also becoming more apparent that vitamin D levels are important to overall health. Vitamin D receptors are found in numerous tissues throughout the body [8], [9]. In humans, adequate levels of vitamin D have been shown to decrease the risk of developing many different conditions, including diabetes, muscular dystrophy, hypertension, and inflammatory bowel disease [8]. When considering the many different physiological functions in vertebrates that are intimately related to vitamin D and calcium metabolism, it should be considered that disease processes in captive species could be related to hypovitaminosis D.

While acquisition and production of vitamin D is highly variable across vertebrate species, unpublished pilot studies have shown that guinea pigs housed indoors have the ability to synthesize vitamin D in the epidermis secondary to exposure to artificial UVB light over the short-term. Since many guinea pigs are housed indoors and not exposed to natural or artificial UVB light, it is possible that these animals may experience chronic vitamin D deficiencies. Inappropriate levels of this essential nutrient and hormone could contribute to common disease processes in these animals such as dental disease, cardiovascular disease, or impaired immune function [4], [5].

While the benefits of UVB exposure for captive animals appear to be strong, it is not without risk. UVB radiation has been associated with the development of skin neoplasia, with the groups at greatest risk including humans with fair complexions and albino or white animals [10], [11]. Direct UVB radiation also has the potential to cause structural damage to the eye at the level of the cornea (keratitis, corneal edema), lens (cataracts), or retina (blindness) [11]. Direct UVB radiation can also cause short-term damage such as photodermatitis and erythema. For these reasons, the safety of UVB supplementation in small mammals should be investigated.

The objectives for this research were to evaluate the clinical and physiologic effects of artificial UVB light supplementation on guinea pigs and to evaluate the long-term safety of artificial UVB light supplementation over the course of six months in these species. The clinical effects being evaluated between the UVB exposed and non-exposed guinea pigs included whether there would be an increased likelihood of ocular disease associated with the cornea, lens, or retina; gross or histologic skin changes; or differences in bone mineralization. The physiologic changes being assessed were related to the 25-hydroxyvitamin D levels, biochemistries, and blood cells. The hypotheses for this study were: 1) animals supplemented with UVB light would produce higher levels of vitamin D than those without supplementation, and that 2) there would be no significant detrimental side effects associated with UVB light supplementation.

## Materials and Methods

### Animals

Twelve juvenile (14-16 weeks), purpose bred, female intact Hartley guinea pigs were used in this study. Hartley guinea pigs are acromelanic albino animals. The pigmentation for acromelanic animals is genetically determined, with slightly variable amounts of pigmentation on the extremities. The animals were obtained from a commercial breeding facility (Charles River Laboratories, Wilmington, MA). Prior to their arrival at the study site, the animals received a standard commercial pelleted feed (Lab Diet, St. Louis, MO) with no additional supplemental vitamin D or UVB light. The animals were allowed to acclimate to their surroundings for 7 days prior to the onset of data collection. This project was approved and performed in accordance with the regulations established by the Institutional Animal Care and Use Committee at the University of Illinois (protocol #13-024).

The animals were housed in 183 cm long×137 cm wide pens. Three animals were housed in each pen. All pens were located in the same room, were enclosed to the ceiling, and the animals were housed on the floor. Room temperature was measured weekly and was kept consistent throughout the study with an average range of 23 to 27 degrees Celsius. Paper shavings (Harlan Laboratories, Indianapolis, IN) were provided as substrate lining the floor of the pen. General room lighting was provided by non-UVB producing ambient fluorescent lighting. The animals were provided with water ad libitum through sipper bottles placed on the front of the pens. All animals were all fed the same weight based diet consisting of Oxbow timothy grass hay (Oxbow Animal Health, Murdoch, NE) and Oxbow Essentials adult guinea pig pellets (Oxbow Animal Health). The vitamin D content of this particular pelleted diet is 900 IU/kg.

### Biochemistry

After one week of acclimation, the animals were anesthetized for baseline sample collection and microchip placement. This sample collection, and all subsequent sample collections, took place between the hours of 3 and 5 pm to correct for natural circadian rhythms and variability. Anesthesia was induced via administration of 5% isoflurane gas (IsoFlo, Abbott Animal Health, North Chicago, IL) and 1 L oxygen via facemask. The animals were maintained on 2–3% isoflurane and 1 L oxygen via facemask after induction. Each guinea pig was placed in dorsal recumbency and 3 ml of blood was collected from the cranial vena cava using a 25 gauge needle attached to a 3 ml syringe. The total blood sample volume was less than 1% of the total body weight of the animal. The samples were placed in lithium heparin microtainers (Benton, Dickinson, and Company, Franklin Lakes, NJ), EDTA microtainers (Benton, Dickinson, and Company, Franklin Lakes, NJ), and glass tubes without anticoagulant (Tyco Healthcare Group LP Mansfield, MA). Avid microchips (Avid Identification Systems, Inc., Norco, CA) were placed subcutaneously in the intrascapular region. The anesthetic gas was turned off, and the guinea pigs were weighed and recovered on supplemental oxygen. Glass tubes without anticoagulant were centrifuged for 15 minutes within 90 minutes of collection. Serum was pipetted and subsequently placed into cryovials and placed in −20 degree Celsius freezer. EDTA samples were submitted to a university clinical pathology laboratory (University of Illinois, Urbana, IL) for complete blood counts and processed using a commercial analyzer (Cell-Dyn3700, Abbott Laboratories, Abbott Park, IL). Complete blood counts were only performed on samples that were not clotted. A commercial chemistry analyzer (Vetscan VS2, Abaxis Inc., Union City, CA) was used to analyze blood chemistries from plasma collected in lithium heparin microtainers within 90 minutes after collection. A mammalian comprehensive diagnostic profile rotor (Abaxis Inc.) was used to measure the biochemistries. This profile quantified levels of albumin (Alb), alkaline phosphatase (ALP), alanine transaminase (ALT), amylase (Amy), total bilirubin (T-bil), blood urea nitrogen (BUN), total calcium (Ca), phosphorus (Phos), creatinine (Creat), glucose (Glu), ionized sodium (Na), ionized potassium (K), total protein (TP), and globulins (Glob). Plasma samples were also submitted on frozen gel packs to a veterinary diagnostic laboratory (Diagnostic Center for Population and Animal Health, East Lansing, MI) for measurement of serum 25-hydroxyvitamin D (25-OHD_3_, radioimmunoassay), ionized calcium (iCa, ion-selective electrode/pH electrode at pH 7.4) and parathyroid hormone (PTH, radioimmunoassay).

### Ophthalmology

Complete ophthalmic examinations under manual restraint were also performed at the initiation of the study. One drop of Tropicamide 1% (Bausch & Lomb Inc., Tampa, FL) was administered into each eye prior to the onset of the exam. The examinations included a neuro-ophthalmic examination, fundic examination, tonometry, slit lamp biomicroscopy (Kowa-SL2, Kowa, Tokyo, Japan), and corneal pachymetry. Tonometry was performed with a rebound tonometer (Tonovet, Icare Finland Oy, Espoo, Finland). The rebound tonometer has three available calibration settings: canine, equine, and “other”: other is used for species for which a calibration table has not been established. The calibration setting for “other” was used in this study. A corneal pachymeter (Compuscan P ultrasonic pachymeter, 20 MHz, Strorz Instrument Company, St. Louis, Missouri) was used in the exam as a gauge of corneal thickness. Pachymetry readings were taken in triplicate for each eye during the exam. Serial ophthalmic examinations were performed at two-month intervals, and at the end of the study period, with the same study procedure repeated for each examination period. A single, blinded investigator (ALL) performed all of the ophthalmic exams.

### Imaging

Computed tomography (CT) scans were performed under sedation at the onset of the study. Computed tomography (CT) of the skull was performed on all subjects using a 16-slice helical CT scanner (GE Lightspeed 16 slice CT Milwaukee, WI, USA) using the following parameters: kVp of 80, mA of 180, slice width of 0.625 mm, pitch of 0.5625, scan field of view 9.6 cm, 512×512 matrix, a rotation time of 0.8 s and using a bone convolution kernel. Each guinea pig was administered a combination of 0.05 mg/kg buprenorphine hydrochloride (Reckitt Benckiser Pharmaceuticals Inc., Richmond, VA) and 0.5 mg/kg midazolam (Hospira Inc., Lake Forest, IL) via intramuscular injection. Once the animal became sedate, it was positioned in sternal recumbency and perpendicular to the gantry with forelimbs positioned caudally. After the scan was complete and the animal recovered from sedation, it was returned to laboratory housing.

**Figure 1:**
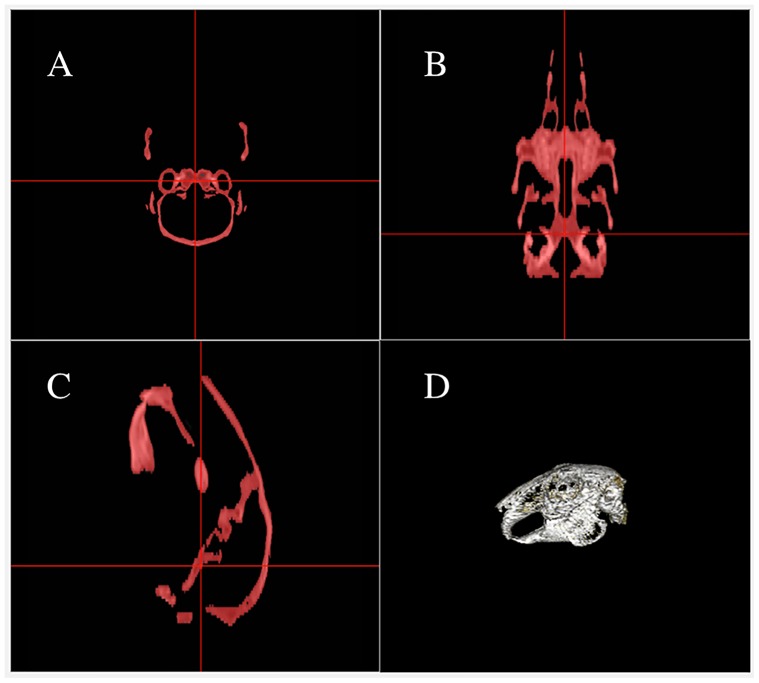
Multiplanar reconstruction view of the bone threshold process using a range of 0 Hounsfield units (HU) – max HU, following initial soft tissue removal. Images A, B and C are the transverse, dorsal and sagittal plane sample images, respectively. Image D is a 3-dimensional volume rendered image of the included bone.

**Figure 2:**
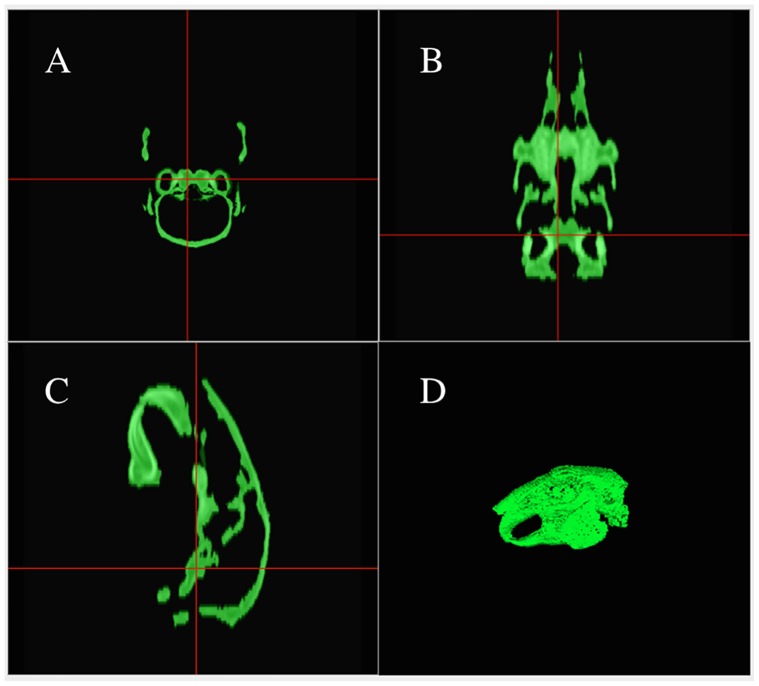
Final window showing the segmented bone and summary of 3D volume parameters: Volume (mL), Mean HU, STD HU, Max HU and Min HU. A-C are the multiplanar reconstruction sample images and the final 3D volume region of interest (D).

Every attempt was made to ensure the head was positioned symmetrically as to not require further post processing reconstruction of the raw data for corrected image planes. The primary transverse CT images included the first two cervical vertebrae and all scans were considered to have good diagnostic quality by a board certified radiologist (SKJ). Segmentation of the skull and teeth was performed using a dedicated DICOM workstation (OsiriX version 5.8.4 64-bit, OsiriX Imaging Software, OsiriX Foundation, Geneva, Switzerland) and segmentation plug-in (Mialite, Center for Medical Image Science and Visualization (CMIV), Linköping University, Sweden) (Fig. 1, Fig. 2). The first step was removing the soft tissue surrounding the skull and neuro tissue within the calvarium and proximal cervical spinal cord. This was achieved by segmentation using a threshold minimum of -100HU, a maximum of 150HU and a smoothing factor of 0.1 [12]. The resulting soft tissue region of interest (ROI) was removed by changing all contained pixels to a value of -2000HU. The segmentation plugin was used again to isolate the skull using a threshold minimum of 0HU, no maximum HU constrains, smoothing factor of 0.1, and a seed (starting point) within the temporal bone. An additional blocker point was used to prevent segmentation of the cervical vertebral bodies. The parameters of the resulting three-dimensional (3D) ROI were recorded for each specimen, such as volume, average attenuation (HU), and attenuation standard deviation.

### UVB Supplementation

After initial sample collection, guinea pigs were assigned to two treatment groups of six animals each using a random number generator (www.random.org). Each group was then randomly divided into two pens housing three animals each. Of the treatment groups, the supplemented (treatment) group was exposed to 12 hours of artificial UVB radiation daily that was controlled by a timer; non-supplemented (control) groups received ambient fluorescent light with no UVB supplementation for 12 hours daily. To provide direct UVB radiation, nine dome lights (Fluker Farms, Port Allen, LA) were suspended above the animals at equidistant intervals, comprising three rows of three lights each. The 5.5 inch domes were anchored to a shelving unit several feet off the ground and suspended directly above the guinea pigs, with no physical barrier present between the animals and light. UVB light was provided using one Oxbow bulb (Oxbow Animal Health, Murdock, NE) in each dome. The domes were suspended 12–14 inches above the ground, in order to maximize exposure to the animals without impeding movement, as well as attempting to mimic the distance that would be used in a typical laboratory or captive cage setting. The amount of UVB radiation and irradiance was measured in microwatts per centimeter squared (µW/cm^2^) by placing a digital UVB meter (Solartech, Inc., Harrison Township, MI) and an irradiance meter (Zoo Med Laboratories Inc., San Luis Obispo, CA) on the ground directly under each bulb, as well as in between the bulbs. The height of the meter was approximately at eye level of the guinea pigs. Measurements were collected weekly, approximately 3 hours after the bulbs were turned on and approximately 3 hours prior to the bulbs being turned off. To correct for UVB degradation over time and provide consistent levels of supplementation, bulbs were replaced if at any time the UVB output from a bulb measured less than 20 µW/cm^2^. The UVB radiation levels measured in the UVB treatment groups ranged from 21–70 µwatts/cm^2^ directly under the lights and 2–19 µwatts/cm^2^ in between the lights. UVB radiation levels in the non-UVB groups were <1 µwatts/cm^2^. The maximum UVB irradiance level in the pens was 3.5, which falls under IV in the UV index spectrum. This value is considered high and equivalent to mid day baskers; however it is well within the safe region, with greater than 7 considered dangerous.

The study was conducted over 6 months. After the UVB light supplementation was initiated, blood samples were collected every three weeks to measure blood chemistries, serum PTH, ionized calcium, and 25-hydroxyvitamin D_3_ levels, for a total of 9 sample collections. Weights were collected during each sample collection. Collection, processing and testing of the blood samples were similar to the techniques described for the samples obtained on day 0. Serial ophthalmologic examinations were performed at two month intervals, and at the end of the study period, for a total of four examinations.

### Histopathologic Evaluation

At the end of the six month period, anesthesia was induced as previously described for initial sample collection. Each animal was placed in dorsal recumbency and a final 3 ml blood sample was collected from the cranial vena cava. While being maintained on 5% isoflurane and 1 L oxygen, 2–3 ml of pentobarbital (Vortech Pharmaceuticals, Dearborn, MI) was administered to the animal via intracardiac injection. Euthanasia was confirmed via auscultation and negative corneal reflex.

CT scans were performed immediately following euthanasia using the identical scanning and positioning parameters described at the beginning of the study. Segmentation of the skull, mandible and teeth was also acquired using the techniques described previously. ROI volume, mean and SD of the attenuations were recorded for each specimen.

A postmortem examination was performed on each animal. A select tissue set was collected from all animals except one animal, which experienced an anesthetic death in which a complete set of tissues was collected. The select tissue set included lung, heart, haired skin- head, haired skin-ear, haired skin-dorsum, haired skin-ventrum, liver, pulmonary artery, aorta, adrenal gland, kidney, duodenum, pancreas, thyroid glands, spleen, esophagus, reproductive tract, trachea, skeletal muscle, eye, and femur. Tissues were fixed in 10% neutral buffered formalin. Following fixation, tissues were embedded in paraffin wax, sectioned at 4 µm, and stained with hematoxylin and eosin. Additionally, histochemical staining with Verhoeff Van-Gieson stain was performed. Histologic lesions were noted and scored using the following categories: no lesions, mild lesions, moderate lesions, and severe lesions. Attempts to characterize apoptosis in the skin, the skin associated with the head, the skin associated with the ears, and the eyes were made to determine if there were any UVB specific injuries caused in these tissues. A single, blinded pathologist (AWS) performed all of the microscopic examinations.

Bone mineral densities (BMD) of guinea pig skulls were measured by dual energy x-ray absorptiometry (Hologic QDR 2000, Marlborough, MA). Prior to analysis, all skulls were preserved and stored at −20 degrees Celsius. For BMD assessment, all skulls were positioned dorsoventrally, and a standardized region of interest (R1) of a constant area (cm^2^) was created to encompass the entire skull size of each specimen. Bone mineral density was measured and expressed as grams of bone per area (gm/cm^2^). The analyst of the bone concentrations was blinded to the study groups (TMF).

### Statistical Analysis

The sample size selected for this study (n = 12; 6 per group) was based on the following assumptions: an expected mean difference in serum 25-OHD_3_ concentrations of 35 nmol/L between treatment and control groups with a SD of 20 nmol/L [13], [14], an alpha of 0.05, and a power of 0.8 (MedCalc 11.3.2.0). Distribution of the data was evaluated by use of the Shapiro-Wilk test. Mean, SD, and minimum-maximum (min-max) values were reported for data that had a normal distribution, whereas the median, 10th to 90th percentiles (%), and min-max values were reported for data that did not have a normal distribution. Data that were not normally distributed were logarithmically transformed in order to perform parametric analysis. If the data was not normally distributed after transformation, appropriate nonparametric statistics were performed.

A linear mixed model was used to evaluate the normally distributed and correlated blood chemistry values, iCa, and 25-OHD_3_ collected in the repeated measures design of the study. This statistical method was selected because one animal died over the course of the study and this type of model can account for missing data. Group and time represented the fixed and random factors, respectively, that were evaluated in the model. The -2 log likelihood was used to determine best fit of the model. Friedman's test was performed on data that was not normally distributed. If significant, Mann-Whitney U tests were performed to determine if a difference existed between groups at each individual time point. A repeated measures ANOVA was used to evaluate differences in Hounsfield units measured on CT scans between groups over time. A repeated measures ANOVA was also used to determine if there was a difference between corneal pachymetry measurements taken in triplicate. Since there was no difference in the corneal pachymetry data, the values were averaged and a linear mixed model was used to assess average corneal pachymetry and intraocular pressure values over time. Levene's test was used to determine if bone mineral density (gm/cm^2^) met the assumption of homogeneity of variance. Because it did, an independent samples t- test was used to determine if bone mineral density differed between groups. Chi square one-tailed tests with a Yates correction or Fisher exact tests were performed to determine if there was any difference in the pathologic findings documented at necropsy between groups. A value of *P*≤0.05 was used to determine significance. Commercial statistical software (SPSS, version 22.0, SPSS Inc, Chicago, IL) and graphpad.com were used to analyze the data.

## Results

Twelve guinea pigs (6 not exposed to UVB, and 6 exposed to UVB) were used in this study. At the initiation of the study the average body weight of the guinea pigs was 497.2 grams (SD: 15.6, min-max: 465.4-520.2 grams), while at the conclusion of the study the mean body weight of the guinea pigs was 946.1 g (SD: 15.6, Min-Max 831-1070); there was no significant difference in body weight noted between groups (F = 0.347, p = 0.557). The mean difference in weight gain from initiation to completion of the study was 449.38 g (SD: 82.1, Min-Max: 349–556) for all animals. Over the study period, weight gain among guinea pigs exposed to supplemental UVB lighting and those not exposed did not differ (t = 0.079, p = 0.938).

Complete blood counts were performed at the beginning and completion of the study to assess the general health of the guinea pigs. Unfortunately, a number of the samples collected at both the beginning and end of the study were clotted and could not be evaluated. Because of the small sample set, statistical comparisons were not performed. However, at least one complete blood count result was available for each guinea pig at either the beginning or conclusion of the study, and all results were within published reference limits [5].

**Figure 3:**
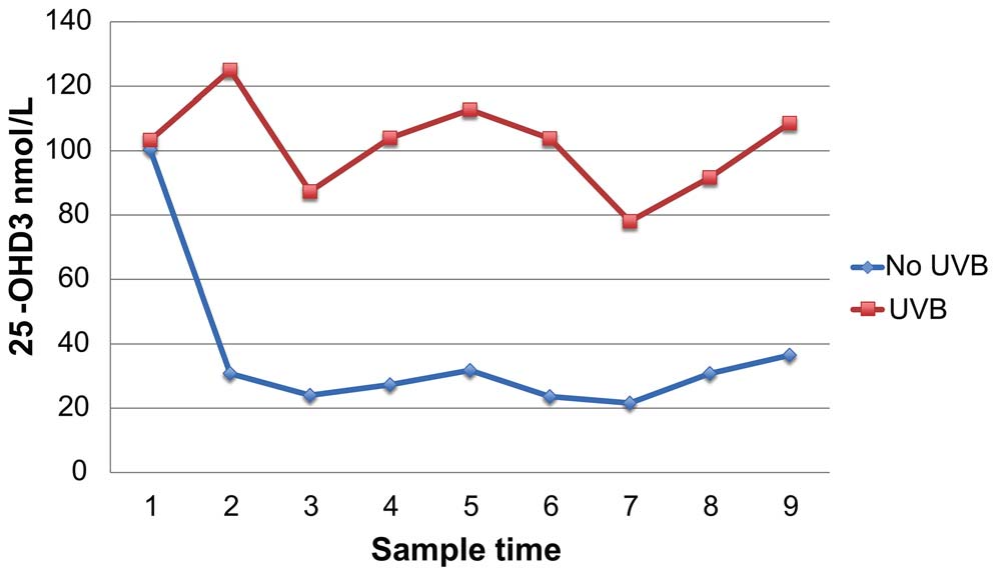
Serum 25-OHD_3_ in guinea pigs between groups over time (sample number).

**Figure 4:**
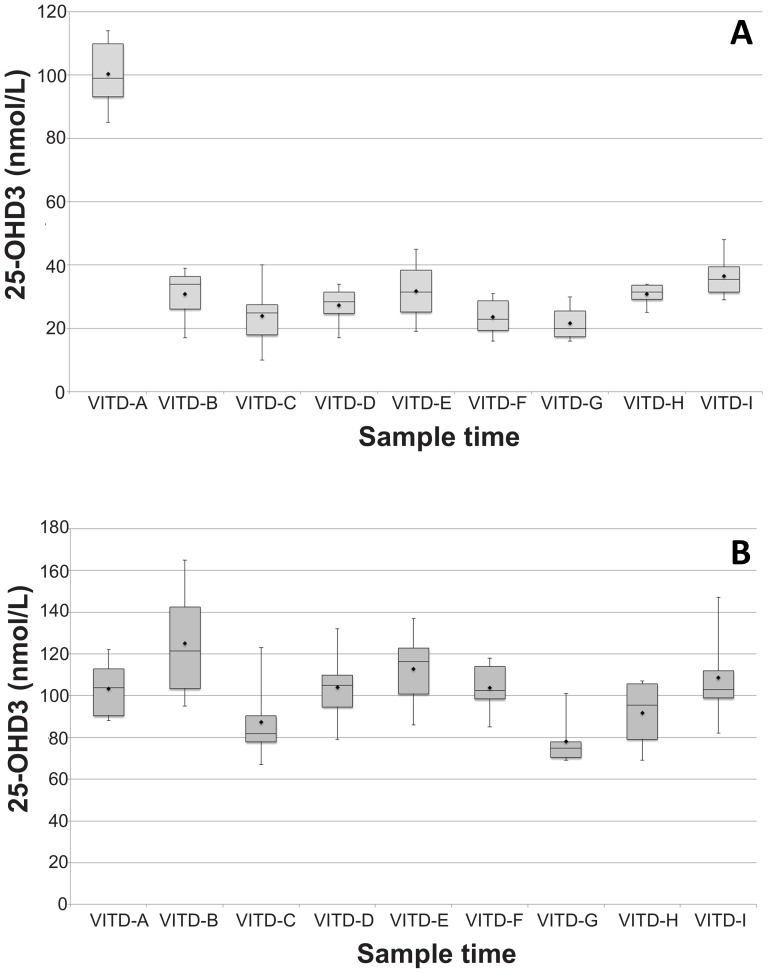
A) Guinea pig serum 25-OHD3 levels with no UVB supplementation. B) Guinea pig serum 25-OHD3 levels for UVB supplemented group. (Time = sample number).

Serum 25-OHD_3_ values differed significantly by group over time (F = 576.126, p<0.0001) (Fig. 3, Fig. 4) with higher values seen in the UVB supplemented group. Since the animals arrived at the University of Illinois facility having been fed a different diet than the study diet, mean, SD, and min-max values including initial baseline data and excluding the baseline data are shown in Table 1. There was no significant difference in the 25-OHD_3_ concentrations between the two pens of guinea pigs exposed to the UVB lights (F = 0.022, p = 0.884) or between the two pens of guinea pigs not exposed to the UVB lights (F = 1.082, p = 0.357). This comparison was done to rule out potential differences in light exposure between treatment groups or quantity of food consumed between control groups.

**Table 1:** Guinea pig serum 25-OHD_3_ values with and without baseline values included by group.

Serum 25-OHD_3_	Mean (nmol/L)	Std Deviation	Range
***With baseline***			
No UVB	36.3333	24.41698	10–114
UVB	101.4906	21.80916	67–165
***Without baseline***			
No UVB	28.3333	8.36236	10–48
UVB	101.2553	22.6816	67–165

When analyzing the blood chemistry values by group and over time, no significant differences were found for PTH, Ca, Phos, calcium/phosphorus ratio (Ca/Phos), K, ALP, ALT, Amy, T-bil, BUN, Creat, Glob, or Gluc values. However, significant differences by group were found for ionized calcium (iCa, F = 117.704, p<0.0001), Na (F = 4448.23, p<0.0001), Alb (F = 8793.077, p<0.0001), and TP (F = 169258.81, p<0.0001). Mean, SD, and min-max values for each group are shown in Table 2.

**Table 2:** Guinea pig biochemical values by group in which statistical significance was found (p<0.0001).

Variable	Mean	SD	Range
***Ionized Calcium*** * (mmol/L)*			
No UVB	1.52	0.07	1.35–1.65
UVB	1.58	0.09	1.29–1.74
***Sodium*** * (mmol/L)*			
No UVB	144.32*	0.38	138–150
UVB	140.19*	0.35	137–148
***Albumin*** * (g/dL)*			
No UVB	4.40	0.28	4.0–5.5
UVB	4.22	0.26	3.7–4.8
***Total protein*** * (g/dL)*			
No UVB	6.08*	0.35	5.0–6.5
UVB	5.79*	0.36	4.6–6.3

*Denotes estimated marginal mean

There was a significant difference in corneal thickness between groups for each eye (right eye [OD]: F = 149.527, p<0.0001; left eye [OS]: F = 30.525, p = <0.0001), with the UVB supplemented group having thicker corneas (OS and OD). Average corneal pachymetry measurements by group are shown in Table 3. There was no significant difference in intraocular pressure noted between groups for either eye (right eye [OD]: F = 0.196, p = 0.66; left eye [OS]: F = 1.1, p =  0.3). No other abnormalities were noted upon examination of the eyes, with minor exceptions. One animal developed a corneal foreign body (OS) from what appeared to be organic material (i.e., bedding or food). Proparacaine was applied to the eye and the foreign body was easily removed with sterile eye wash using gentle lavage. Fluorescein stain was applied, and the cornea was then observed via slit lamp to have a mild superficial ulcer and keratitis, secondary to the corneal foreign body. The animal was administered one treatment of triple antibiotic ointment in that eye and rechecked in 48 hours. At that time the keratitis had completely resolved and no further treatment was necessary. Another animal developed serous ocular discharge OS with no associated conjunctivitis, which resolved within 48 hours without treatment. Both of these animals were in the UVB supplementation group.

**Table 3:** Guinea pig corneal pachymetry values for each eye by group (p<0.0001).

Eye	Mean (µm)	Std Deviation	Range
***Right (OD)***			
No UVB	245.72	14.16	212–269
UVB	257.62	21.35	213–288
***Left (OS)***			
No UVB	242.64	13.40	216–268
UVB	262.77	21.95	213–295

For the initial CT exams, the mean Hounsfield units were 1076.478 (SD: 32.8606, Min-Max1015.18-1126.14); for the final exams the mean HU was 1208.003 (SD: 44.360, Min-Max 1152.64-1293.49). There was a significant increase in Hounsfield units noted on the CT images over time (F = 173.414, p = <0.0001) but not between control and UVB supplemented groups (F = 0.559, p = 0.472). There was no evidence of dental disease on the CT scans. There also was no significant difference noted in the bone mineral density between the two groups obtained via DEXA scan (t  =  −0.713, p = 0.494).

**Figure 5:**
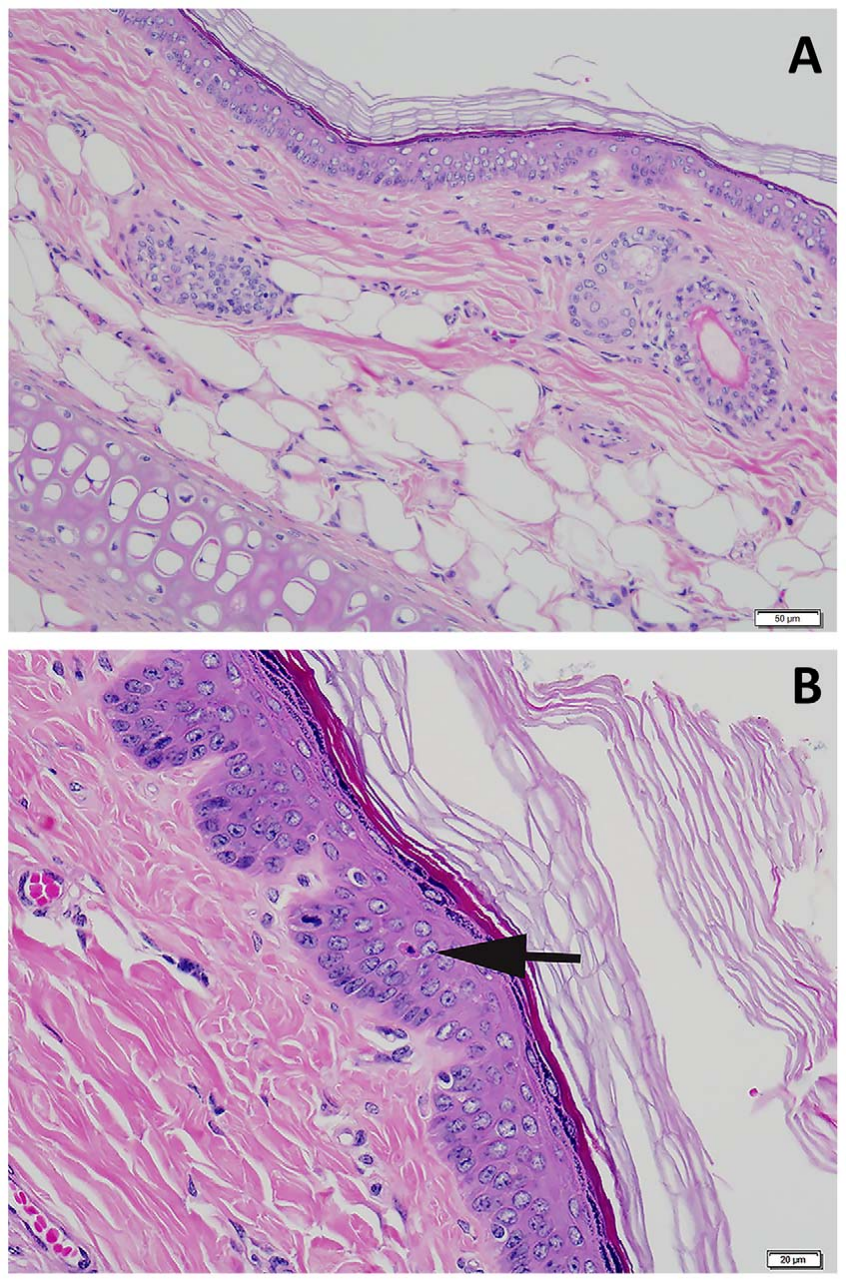
A. Haired skin, ear. Microscopically normal skin from the ear. B. Haired skin, ear. Rare keratinocyte apoptosis within the stratum spinosum. Apoptotic keratinocyte denoted with arrow. Hematoxylin and eosin stain.

**Figure 6:**
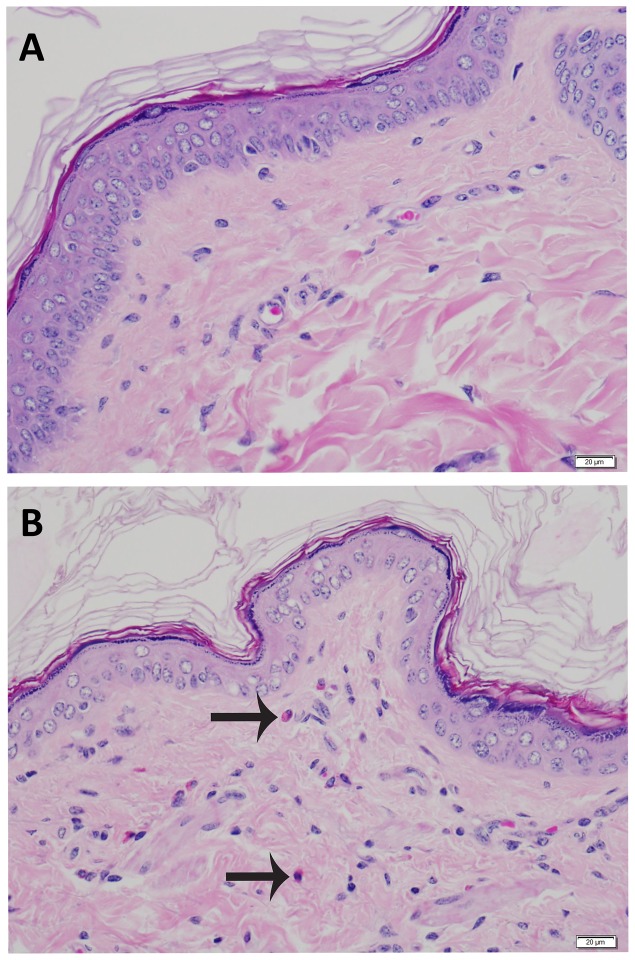
A. Haired skin, head. Microscopically normal skin from the head. B. Haired skin, head. Increased cellularity of the dermis by small numbers of lymphocytes and eosinophils. Arrows denote eosinophils. Hematoxylin and eosin stain.

**Figure 7:**
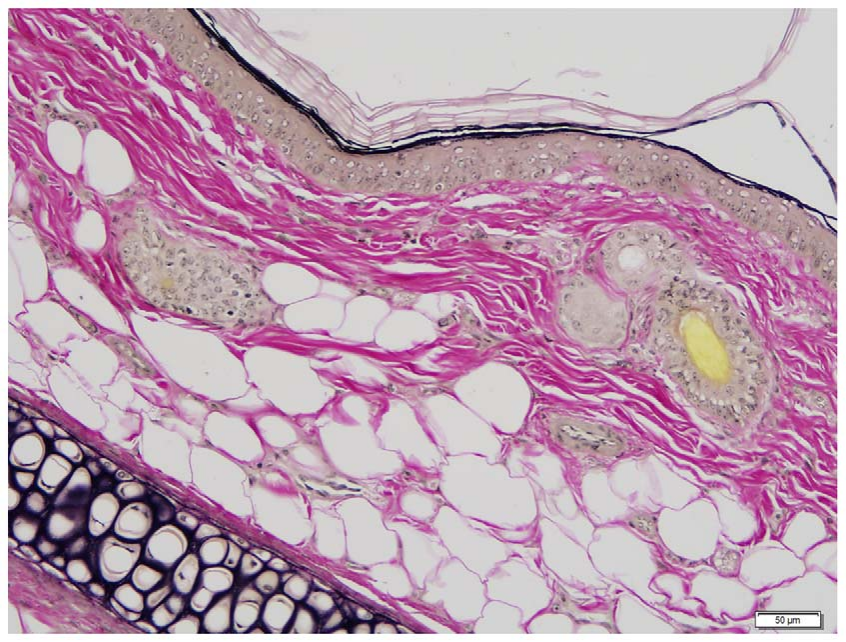
Haired skin, ear. Microscopically normal skin from the ear. Note the lack of positive (black) staining within the dermis. Verhoeff Van-Giessen stain.

No significant differences were found when comparing necropsy lesions between groups (all p>0.05) (Table 4). Mild dermatologic changes were noted equally across groups consisting of rare suprabasilar apoptotic keratinocytes, small numbers of lymphocytes, plasma cells, and eosinophils scattered in the superficial dermis, and minimal to mild epidermal hyperplasia on skin from the ears (Fig. 5) or head (Fig. 6). No elastin abnormalities were noted on Verhoeff-Van Gieson staining (Fig. 7). Multifocal and mild vacuolar hepatopathy (glycogen) were also noted equally across groups. The only mineralization documented was multifocal and mild mineralization within renal cortical tubules in a guinea pig with no UVB supplementation, and a single mineralized muscle myofiber in the skeletal muscle of a guinea pig with UVB supplementation.

**Table 4:** Guinea pig lesions found on necropsy by organ.

Organ	No UVB		UVB	
	n	percentage	n	percentage
Liver	3*	50%	4#	66.7%
Kidney	1	16.7%	0	
Haired Skin- Ear	2	33.3%	1#	16.7%
Haired skin- Head	1*	16.7%	0	
Haired skin- Ventrum	1	16.7%	0	
Esophagus	1*	16.7%	0	
Trachea	1*	16.7%	2#	33.3%
Femur	1*	16.7%	0	
Lung	0		2#	33.3%
Spleen	0		1#	16.7%
Eye	0		1#	16.7%
Muscle	0		1	16.7%

*Denotes all lesions were documented in the same animal.

# All lesions documented in the same animal. This animal died post-anesthetic recovery.

## Discussion

Findings from the present study demonstrated that guinea pigs provided artificial supplementation of UVB light produced and maintained significantly higher serum 25-OHD_3_ levels compared to those without UVB supplementation. In many vertebrates, vitamin D_3_ (cholecalciferol) can be synthesized after exposing the skin to UVB radiation. This initiates the photobiochemical conversion of the precursor 7-dehydrocholesterol in the dermis to previtamin D_3_, which undergoes isomerization into vitamin D_3_ and is hydroxylated to 25-OHD_3_ in the liver [15]–[17]. 25-OHD_3_ is the storage form of vitamin D, and therefore is considered the best measure of vitamin D status in the body [18], [19]. When vitamin D is needed systemically, the kidneys are responsible for conversion of 25-OHD_3_ to 1,25(OH)_2_D_3_, although conversion has been demonstrated in other tissues as well. The findings in this study, along with a previous short term pilot study, reinforce that guinea pigs have the ability to generate significant quantities of vitamin D through UVB exposure, despite adequate levels being provided in the diet, and thus may be an important conserved evolutionary mechanism not currently utilized in captive guinea pig husbandry practices.

While a significant difference was noted between groups over time, reference ranges for optimum serum 25-OHD_3_ in wild or captive guinea pigs have not been established. In humans, the mammal in which vitamin D deficiency is most commonly diagnosed and treated, a serum 25-OHD_3_ concentration of <50 nmol/L is considered deficient [6]. However, it should be noted that this minimum value is a general recommendation by most experts, and a general consensus on optimum levels of 25-OHD_3_ in humans does not exist. Since guinea pigs and humans share similar methods of vitamin D acquisition, both utilizing UVB exposure and diet, it might be tempting to extrapolate the guinea pig values to human reference ranges. Based on the current results, the group not supplemented with UVB light (28.33±8.36 nmol/L, range 10–48) would be considered chronically deficient in vitamin D. However, such a direct comparison is difficult because of differences in physiology between our two species. Humans are omnivores that are capable of deriving vitamin D from animal and fortified sources in their diet, in addition to the vitamin D they can obtain from the plant material in their diet, as in the case of guinea pigs. On the other hand, guinea pigs have evolved in a high altitude, low latitude environment where exposure to UVB radiation would be greater than that which humans evolved. While it may appear as though both species are adapted to synthesize and maintain vitamin D levels under these different conditions, evaluating the two different methods for which they derive the vitamin D is important to consider. Dietary sources of vitamin D tend to persist in the body, while vitamin D produced via photoconversion can be turned on or off based on need. In theory, guinea pigs may be adapted to lower levels than humans, but the results of the current study suggest otherwise since animals exposed to UVB maintained 25-OHD_3_ levels well over 50 nmol/L.

An important finding in this study was that, over a six-month period, prolonged exposure of guinea pigs to artificial UVB light sources led to a statistically significant increase in serum 25-OHD_3_ levels that were sustainable over time. This ability to sustain substantially higher levels of 25-OHD_3_ from UVB exposure compared to animals provided vitamin D in diet alone reinforces that these animals evolved with this mechanism to procure and maintain adequate levels of vitamin D. Guinea pigs are naturally a high altitude, low latitude species, with ample access to sunlight and natural UVB radiation in their home range. A recent pilot study also demonstrated that chinchillas (*Chinchilla lanigera*), which are also native to the Andes Mountains, have the ability to produce 25-OHD_3_ through photobiochemical synthesis following exposure to artificial UVB lights [14]. Other animals native to similar environments, such as llamas (*Lama glama*) and alpacas (*Vicugna pacos*), have been shown to develop significant disease secondary to a seasonally dependent hypovitaminosis D [20]. When comparing the vitamin D levels of the guinea pigs in this study to rabbits maintained in a similar study, the average 25-OHD_3_ level in guinea pigs was higher, despite similar exposure. This could suggest these animals have a higher vitamin D requirement in relation to their natural habitat being at a higher altitude and lower latitude.

An interesting observation in this study was that the baseline serum 25-OHD_3_ values were most similar to the group supplemented with UVB light, and contrary to what was expected, actually decreased after initial baseline collection in the group not supplemented with UVB. Unfortunately, the initial baseline sample collection after arrival at the University of Illinois took place three months prior to the first sample collection post-UVB exposure because of logistics, so it is not known how long it took for the decline in serum 25-OHD_3_ values to occur. During this three-month period, the guinea pigs were fed the same diet and housed under the conditions described previously. After contacting the breeding facility it was determined that, prior to acquisition at the University of Illinois, the animals were fed a pelleted guinea pig feed (LabDiet, St. Louis, MO) that contained a higher concentration of vitamin D (3400 IU/kg), in comparison to the diet fed throughout the time they were housed for the study which contained 900 IU/kg of vitamin D. The 3.8 times higher dietary concentration seen in the diet offered at the breeding facility is almost in direct agreement with the higher mean levels of serum 25-OHD_3_ demonstrated between groups after the initial baseline collection (3.6 times). This finding reinforces that guinea pigs indeed have the capability of increasing their serum 25-OHD_3_ levels via either dietary ingestion or photobiochemical conversion, and also may hint towards optimum serum 25-OHD_3_ maintenance levels in guinea pigs. However, how they acquire vitamin D could be important, especially as it relates to the potential development of hypervitaminosis D. The dietary vitamin D requirements for guinea pigs are not known, but it is suggested to be 1000 IU/kg diet [21]. Under these guidelines, the commercial diet fed throughout the study adheres to those recommended requirements. The formula used at the breeding facility was 3.8 times higher than this and could, long term, lead to problems as dietary sources are more likely to be attributed to cases of hypervitaminosis D.

When evaluating circulating levels of 25-OHD_3_, one important factor to consider is that systemic mineralization has been observed due to hypervitaminosis D secondary to increased levels of vitamin D in the diet via clinical or experimental report [22]. Toxicity was described in a cohort of guinea pigs fed 150 times the normal concentration of vitamin D in their diet, and subsequently they developed systemic disease with lesions consisting of renal interstitial fibrosis with tubular mineralization, soft tissue mineralization in multiple organs, hepatic lipidosis, and pneumonia [22]. Serum 25-OHD_3_ levels were obtained in two animals (445 nmol/L and 541 nmol/L), both of which would be considered toxic if using human guidelines [6]. There have been no reports of hypervitaminosis D secondary to photoconversion of 7-DHC. This is likely because photobiochemical conversion of 7-DHC to previtamin D_3_ to hydroxylation in the liver to 25-OHD_3_ is tightly regulated within the body. In humans, mechanisms have been elucidated which have shown sunlight actually destroys excess previtamin D_3_ or vitamin D_3_ when exposure is prolonged or excessive [23]. Previtamin D_3_ can revert to the parent compound, 7-DHC, or other biologically inert metabolites, to avoid potentially harmful accumulation of circulating vitamin D in the body [24]. Since other pathways involving photoconversion in guinea pigs are similar to the human, it is highly likely that these control mechanisms exist in the guinea pig as well; however, specific investigation in guinea pigs is warranted.

Ionized calcium values were found to be significantly increased in the group supplemented with UVB radiation, while total protein and albumin were significantly higher in the non-UVB supplemented group. Vitamin D_3_ is essential for active calcium absorption in the intestine, as well as maintaining serum calcium levels in most vertebrates. In the blood, calcium can be free (iCa), bound to proteins, or complexed with other molecules [25]. Ionized calcium is the most important indicator of calcium status, since it is the major active form in the body. It is also typically tightly regulated, as small increases can lead to cell dysfunction. In women, intestinal calcium transport increased by 45–65% when 25-OHD_3_ levels were increased from 50 to 72 nmol/L [26]. Although biologically within normal limits, this statistically significant increase in the guinea pigs represents a trend in available active calcium, secondary to increased circulating vitamin D. In the non-UVB supplemented group, the higher albumin and total protein in combination with the lower iCa and 25-OHD_3_ suggest that these animals have lower availability to active calcium. These findings are important because they reinforce that vitamin D plays an important role in calcium homeostasis for guinea pigs. While the levels obtained were within reference ranges, the dynamic nature of ionized calcium suggests that interpreting its meaning from single reference points could underestimate its true value. Follow-up longitudinal studies evaluating the relationship of iCa and 25-OHD_3_ in clinically normal and diseased guinea pigs are needed.

In children, chronic vitamin D deficiency can lead to developmental abnormalities such as failure of attaining peak height or bone mineral density, pathologic fractures, poor growth, and slow development. Adults with vitamin D deficiency are at an increased risk for osteopenia, osteoporosis, and fracture [8]. Effects of hypovitaminosis D have also been observed in other animals in experimental studies, including rabbits and dogs [27]–[29]. The animals used in this study were juveniles, and thus it might be expected that those not supplemented with UVB radiation would experience decreased bone mineral density or poor growth if deficient in vitamin D during this vital growth period. However, due to humane reasons, levels in the diet were not restricted and the amount provided should have been sufficient based on current recommendations. No significant difference in Hounsfield units measured on CT scans was observed between groups; however, a significant increase was noted over time in both groups. This is consistent with normal growth and bone development patterns. Also, there was no difference in the bone mineral density of the skulls between groups noted at the end of the study. These findings indicate that no adverse effects to the bone were noted during the growth phase of these animals, regardless of UVB exposure. Because these animals were juvenile animals that were only observed until approximately one year of age, it is not likely we would witness any effects secondary to chronic vitamin D deficiency. Dental disease, which has been hypothesized to be due to chronic vitamin D deficiency, often involves resorption of alveolar bone and also often occurs progressively over time [30], [31]. The results of this study do confirm that a dietary source of vitamin D at 900 IU/kg during development is sufficient for normal skeletal growth. Future studies that follow guinea pigs longitudinally will be important to determine whether animals exposed to UVB, and subsequently maintain higher levels of circulating 25-OHD_3_, do so to minimize the potential for developing specific pathologies.

Corneal thickness was found to be significantly increased in guinea pigs exposed to UVB radiation; this group also had higher circulating levels of 25-OHD_3_. Although increased corneal thickness may be a normal aging change, a difference was identified between groups over time. The phenotype, diet, and environmental factors were all the same between groups with the exception of the UVB supplementation and there was no significant difference in weight between groups over time. Due to these factors, we can attribute the increase in corneal thickness to the UVB radiation. No evidence of keratitis that could be associated with UV toxicity was identified during slit lamp examinations, or on histopathology. Corneal thickness in both groups was slightly increased in comparison to another study, which found an average thickness of 227.85±14.09 µm [32]. Since no ocular pathology was identified in any of the eyes, it is possible that the increase in corneal thickness may be a protective mechanism to limit the potential for damage to internal structures of the eye such as the lens and retina. Cataracts are the most common pathology reported in the eyes of vertebrates exposed to unhealthy levels of ultraviolet radiation (UVA) [33]. However, it has been demonstrated that the cornea provides a significant filter to the amount of UV radiation that reaches the lens [34]. A study that placed UV protective contacts on guinea pig eyes found increased damage to corneas without UV protective contacts [35]. In this high altitude species, it is possible the cornea has developed protective mechanisms over time. The cornea has also been shown to have the ability to synthesize vitamin D in a similar fashion to that of the skin [36]. It has been demonstrated that increased vitamin D levels enhance the epithelial barrier function of the cornea [37].

No significant differences were noted in the gross necropsy or histopathologic findings between study groups. However, there were lesions recorded in individual animals that are noteworthy. The most common lesion reported was multifocal to diffuse and mild vacuolar hepatopathy (glycogen) (7/12, 58.3%). Vacuolar hepatopathy is commonly associated with administration of corticosteroids, or excessive physiologic release of steroids (i.e. hyperadrenocorticism). Since guinea pigs are a prey species, excess corticosteroids may naturally rise as a response to stress, leading to these types of histologic changes. In humans, individuals with fair skin and fair hair are at higher risk of developing neoplasia secondary to UVB exposure [11], and the same has been shown for white cats [10]. In this study, albino animals were used because it was expected that they would be most sensitive to UVB exposure and at the greatest risk for potential adverse side effects secondary to UVB exposure. Presence of rare suprabasilar apoptotic keratinocytes were noted in three cases, with more noted in animals without UVB supplementation than with UVB. Typically, apoptotic keratinocytes are associated with UV exposure. In humans, it is well documented that UV radiation induces DNA damage, oxidative stress, and inflammatory processes in keratinocytes, which can result in photocarcinogenesis [38]. When apoptotic keratosis is noted in small amounts, it is likely an indication of a defense mechanism network that has been developed to control potentially harmful environmental contaminants. Damage to skin collagen and elastin is a hallmark of long term exposure to solar UV radiation in humans [39]. Verhoeff-Van Gieson staining was performed to detect elastin abnormalities that may be present in conjunction with solar elastosis [40]; however, no elastin abnormalities were noted in either group. This finding, in addition to the lack of ocular pathology, suggests that the UVB levels and irradiance used in this study are not toxic in guinea pigs over an extended period (6 months).

## Conclusions

The results of this study indicate that guinea pigs have the ability to increase their 25-OHD_3_ levels via photobiochemical production, and that the utilization of this pathway is sustainable over a six month period. Ionized calcium had a positive association with increased serum vitamin D levels, while albumin and total protein had an inverse relationship. Corneal thickness increased in animals exposed to UVB light; with no ocular pathology noted, this may be indicative of a protective mechanism. This study also showed that there were no apparent detrimental health effects associated with artificial UVB exposure as characterized by complete blood counts, serum biochemistry analyses, ophthalmologic examinations, CT scans, a DEXA scan, and necropsy and histopathology. While it is known that serum 25-OHD_3_ levels can also be increased via dietary supplementation alone, as evidenced by the baseline data in this study, this method of obtaining vitamin D is not necessarily benign. Hypervitaminosis D and associated lesions have been documented secondary to excess oral vitamin D supplementation. Including the findings of this study in guinea pigs, no reports of vitamin D toxicosis have been documented secondary to UVB supplementation in any vertebrate. In conclusion, the results of this study suggest that artificial UVB supplementation is a safe and effective method for increasing and maintaining vitamin D levels in guinea pigs, and that individuals responsible for managing these animals in captivity should consider providing UVB exposure to the animals in their care.
